# Paliperidone LAI-Induced Leukocytopenia: A Case Report

**DOI:** 10.1192/j.eurpsy.2024.1529

**Published:** 2024-08-27

**Authors:** K. Laškarin, K. Matić, S. Vuk Pisk, M. Grah, V. Grošić, I. Filipčić

**Affiliations:** ^1^University Psychiatric Hospital “Sveti Ivan”, Zagreb; ^2^Faculty of Dental Medicine and Health “Josip Juraj Strossmayer” University of Osijek, Osijek; ^3^School of Medicine, University of Zagreb, Zagreb, Croatia

## Abstract

**Introduction:**

Antipsychotics effectively manage psychotic symptoms but may have side effects. Patients with schizophrenia often lack insight into their condition, leading to nonadherence. Long-acting injectable (LAI) antipsychotics aim to overcome this, reducing relapse risks. Paliperidone LAI, a second-generation antipsychotic, has a lower side effect profile when compared to first-generation counterparts. Blood dyscrasias, like neutropenia and lymphopenia, increase infection susceptibility. This case report describes an instance of leukocytopenia arising during paliperidone LAI treatment, which quickly resolved after the discontinuation of the medication.

**Objectives:**

This case report describes an instance of leukocytopenia arising during paliperidone LAI treatment, which quickly resolved after the discontinuation of the medication.

**Methods:**

**Results**

CASE

A 42-year-old female with schizophrenia, nonadherent to previously prescribed medication was admitted to our acute psychiatric department. She experienced positive symptoms (paranoid delusions), as well as disorganized thinking and behavior. Oral risperidone 4 mg two times a day was recommenced and titrated with mild improvement in her psychotic symptoms with the idea of switching to paliperidone LAI and eventually ceasing oral medication. Oral paliperidone was unavailable for prescription due to local restrictions. At admission her routine laboratory tests showed no abnormalities, but 5 days after receiving paliperidone LAI, routine laboratory tests showed a strong decrease in her WBC and absolute neutrophilic and lymphocytic count ( Lkc 2.89x10^9^/L, Neut 1.57x 10^9^/L, Lym 0.88x 10^9^ /L ). Antipsychotic-induced blood dyscrasia was suspected and paliperidone depot was discontinued. The patient had rapid improvement in her WBC reaching the reference range in 10 days ( Lkc 4.23 x 10^9^/L, Neut 2.51x10 ^9^/L, Lym 0.98x 10^9^/L). Sertindole was introduced consdering her history of a good therapeutic response to the drug, with improvement in psychotic symptoms. She is currently stable taking sertindole 16 mg/day, clonazepam 2 mg/day and alprazolam 0.5 mg/day.

DISCUSSION

The onset of neutropenia and lymphopenia post-paliperidone LAI initiation, resolving in 10 days,, indicate a direct association. Few cases report to date describe paliperidone-induced leukocytopenia, with rapid recovery post-discontinuation. Proposed mechanisms include bone marrow suppression and peripheral WBC destruction. It has been proposed that drug-induced neutropenia is often dose-dependent, which could explain why our patient exhibited tolerability to risperidone but developed cytopenia upon transitioning to depot paliperidone.

**Conclusions:**

While cases of agranulocytosis have been reported in association with the use of other antipsychotics these antipsychotics do not require the same monitoring as clozapine. Our case emphasizes the need for vigilant blood dyscrasia monitoring during antipsychotic therapy.

**Disclosure of Interest:**

None Declared

